# Automated Lumen Segmentation in Carotid Artery Ultrasound Images Based on Adaptive Generated Shape Prior

**DOI:** 10.3390/bioengineering11080812

**Published:** 2024-08-09

**Authors:** Yu Li, Liwen Zou, Jiajia Song, Kailin Gong

**Affiliations:** 1 School of Mathematics and Statistics, Nanjing University of Science and Technology, Nanjing 210094, China; liyu_qian@njust.edu.cn; 2 Department of Mathematics, Nanjing University, Nanjing 210093, China; dz20210008@smail.nju.edu.cn; 3 Affiliated Nanjing Brain Hospital, Nanjing Medical University, Nanjing 210029, China; homyee@163.com

**Keywords:** shape prior, lumen segmentation, carotid artery, variational model, ultrasound image

## Abstract

Ultrasound imaging is vital for diagnosing carotid artery vascular lesions, highlighting the importance of accurately segmenting lumens in ultrasound images to prevent, diagnose and treat vascular diseases. However, noise artifacts, blood residue and discontinuous lumens significantly affect segmentation accuracy. To achieve accurate lumen segmentation in low-quality images, we propose a novel segmentation algorithm which is guided by an adaptively generated shape prior. To tackle the above challenges, we introduce a shape-prior-based segmentation method for carotid artery lumen walls. The shape prior in this study is adaptively generated based on the evolutionary trend of vessel growth. Shape priors guide and constrain the active contour, resulting in precise segmentation. The efficacy of the proposed model was confirmed using 247 carotid artery ultrasound images, with experimental results showing an average Dice coefficient of 92.38%, demonstrating superior segmentation performance compared to existing mathematical models. Our method can quickly and effectively perform accurate lumen segmentation on low-quality carotid artery ultrasound images, which is of great significance for the diagnosis of cardiovascular and cerebrovascular diseases.

## 1. Introduction

Cerebrovascular disease frequently occurs as a result of conditions such as atherosclerosis, hypertension or thrombosis, making it the third leading cause of death worldwide [1,2]. The occurrence of ischemic stroke is strongly associated with the rupture of atherosclerotic plaques in the carotid arteries and their dissection. Rupture can result in significant vascular blockage due to the distal spread of thrombosis [3,4].

Ultrasonic imaging offers numerous advantages, such as simplicity of operation, cost-effectiveness, non-invasiveness, safety, real-time display and easy detection repeatability, making it the preferred method for examining cervical artery disease [5]. It provides detailed views of blood vessels’ anatomical structure and plaque characteristics, as well as metrics like blood flow rate and vascular stenosis. However, generating high-quality images for segmentation through ultrasonic imaging remains a significant challenge due to speckle noise, plaque formation, variations in blood flow dynamics and ultrasound artifacts. The similar echogenicity between the lumen and surrounding tissues, as well as complex anatomy, further complicate accurate segmentation. Additionally, manual segmentation of the lumen is demanding and time-consuming, requiring a high level of recognition and specialized knowledge of carotid artery anatomy.

It is crucial to accurately segment the vessel lumen in carotid artery ultrasound images to detect and monitor cardiovascular diseases, particularly atherosclerosis, a leading cause of strokes. Precise delineation of the arterial boundaries enables the assessment of atherosclerosis severity by measuring lumen size and identifying plaque buildup [6,7,8]. This information allows clinicians to predict stroke risk better, personalize patient treatment plans and track disease progression [9,10]. As a result, there is a high demand for fast, reproducible and comprehensive diagnostic techniques [11,12]. Despite the progress in segmentation methods for carotid artery analysis, several challenges persist, affecting the accuracy of these measurements [13,14,15,16]. These challenges include a need for more consideration for the shape of the carotid artery in many current methods, which often focus solely on the visual appearance of images and overlook prior information about the artery’s shape. This oversight can lead to inaccurate estimates, ultimately diminishing the accuracy and stability of the segmentation process. Another issue area for improvement is the susceptibility of commonly used techniques to various image quality problems, such as speckle noise, low contrast, grayscale non-uniformity and boundary discontinuities.

In our study, we propose a new approach to address the challenges that current methods face for analyzing carotid artery images. We introduce a model-driven method that uses anatomically generated shape priors to accurately segment the lumen walls in longitudinal carotid artery ultrasound images. In the first stage, each two-dimensional image is cropped to remove text and annotations and extract the region of interest. This reduces the image area and helps minimize the detection of unexpected tissue structures that could affect the detection of the lumen wall. Cropping the images also improves the computational efficiency in segmentation tasks. In the second stage, we apply a variational model with anatomically generated shape priors to achieve accurate segmentation results. The shape prior information helps overcome disturbances such as edge loss and blurred target edges, ensuring that contours maintain an appropriate shape. The active contour leverages edge information and shape prior to effectively approximating the actual boundary of the lumen wall, thus achieving more precise segmentation of the lumen wall in low-quality images.

## 2. Method

### 2.1. Preliminary

#### 2.1.1. The Chan–Vese Model

The principle of the variational segmentation model can be summarized as follows: a curve is defined within the image, affected by both internal and external energy, which are related to the intrinsic characteristics of the target contour and target features, respectively. The properties of this curve (also referred to as the active contour) and its movement are described with an energy function derived from physical motion systems. The goal is to optimize this energy function so that the energy of the curve gradually diminishes as it moves, ultimately coming to rest near the desired target contour. Chan and Vese introduced a region-based variational model called the CV model [17]. It is built on the Mumford–Shah (M-S) model [18] and Level Set Function (LSF) assuming that image intensity is uniform. Its fundamental concept involves utilizing the global intensity difference between inner and outer average strengths $c_{1}$ and $c_{2}$, separated by a closed curve *C*, to propel the evolving curve towards the target boundary. The energy function is defined as follows:

(1) $$\begin{array}{cc} E_{CV}\left(c_{1},c_{2},\phi \right)=\; & \mu \int_{\Omega }\delta \left(\phi \left(x,y\right)\right)\left|\nabla \phi (x,y)\right|\;dx\;dy+\nu \int_{\Omega }H\left(\phi \left(x,y\right)\right)\;dx\;dy\\ \; & +\lambda_{1}\int_{\Omega }{\left|I-c_{1}\right|}^{2}H\left(\phi \left(x,y\right)\right)\;dx\;dy\\ \; & +\lambda_{2}\int_{\Omega }{\left|I-c_{2}\right|}^{2}\left(1-H\left(\phi \left(x,y\right)\right)\right)\;dx\;dy \end{array}$$

where $\lambda_{1}>0,\;\lambda_{2}>0$, μ≥0 and ν≥0 are fixed parameters, *I* is a given image in the domain Ω, *H* is the Heaviside function and δ(ϕ(x,y)) is the derivative of *H*. Usually, the first two and the last two terms in Equation (1) are called the regularization term and the data term, respectively.

To address the minimization problem, the Euler–Lagrange equations were applied to describe the energy function in Equation (1). However, the traditional CV model associated with this approach exhibits two significant drawbacks: (i) Inability to handle intensity inhomogeneities: The model struggles with images characterized by intensity variations, such as noise, blurred boundaries and low contrast. These factors can severely impair the model’s ability to segment images accurately. (ii) Re-initialization issues with the LSF: Regular re-initialization of the LSF is necessary to preserve the sign distance information. This not only adds substantial computational overhead but also unpredictably impacts numerical accuracy. The continuous requirements to recalibrate the LSF can lead to inefficiencies and potential inaccuracies in the segmentation process.

#### 2.1.2. The Distance Regularization Level Set Evolution Model

To address the above issues, Li et al. [19] proposed the DRLSE model. DRLSE has the inherent ability to maintain the regularity of the LSF, which leads to unique forward and backward diffusion effects to maintain the desired sign distance property, thereby ensuring the stability of level set evolution and the accuracy of LSF and its derivative calculation. Let *I* be an image on the domain Ω, and its energy be defined as

(2) $$\begin{array}{c} E_{DRLSE}\left(c_{1},c_{2},\phi \right)=\;\mu {ℜ}_{p}\left(\phi \left(x,y\right)\right)+\lambda L_{g}\left(\phi \left(x,y\right)\right)+\alpha A_{g}\left(\phi \left(x,y\right)\right) \end{array}$$

where μ>0, λ>0 and α∈R are the coefficients of the energy terms ${ℜ}_{p}\left(\phi \left(x,y\right)\right),L_{g}\left(\phi \left(x,y\right)\right)$ and $A_{g}\left(\phi \left(x,y\right)\right)$, which are defined by

(3) $$\begin{array}{c} {ℜ}_{p}\left(\phi \left(x,y\right)\right)≜\int_{\Omega }p\left(\left|\nabla \phi (x,y)\right|\right)\;dx \end{array}$$

where *p* is a potential (or energy density) function p:[0,∞)→ℜ,

(4) $$\begin{array}{c} L_{g}\left(\phi \left(x,y\right)\right)≜\int_{\Omega }g\delta \left(\phi \left(x,y\right)\right)\left|\nabla \phi (x,y)\right|dx, \end{array}$$

and

(5) $$\begin{array}{c} A_{g}\left(\phi \left(x,y\right)\right)≜\int_{\Omega }gH\left(-\phi \left(x,y\right)\right)dx. \end{array}$$

The *g* in Equations (4) and (5) is the edge-detection function [20], defined as

(6) $$\begin{array}{c} g≜\frac{1}{1+{\left|\nabla G_{\sigma }*I\right|}^{2}} \end{array}$$

where $G_{\sigma }$ is a Gaussian kernel with a standard deviation σ. The convolution in (6) is used to smooth the image to reduce the noise. This function *g* usually takes smaller values at object boundaries than that at other locations. δ and *H* are the Dirac delta function and the Heaviside function, respectively. In practice, these two functions in the functionals $L_{g}$ and $A_{g}$ are approximated by the following smooth functions $\delta_{\varepsilon }$ and $H_{\varepsilon }$ as in many level set methods, defined by

(7) $$\begin{array}{c} \delta_{\varepsilon }\left(x\right)=\left\{\begin{array}{cc} \frac{1}{2\varepsilon }\left[1+\cos\left(\frac{\pi x}{\varepsilon }\right)\right], & \left|x\right|\leq \varepsilon \\ 0, & \left|x\right|>\varepsilon \end{array}\right. \end{array}$$

and

(8) $$\begin{array}{c} H_{\varepsilon }\left(x\right)=\left\{\begin{array}{cc} \frac{1}{2\varepsilon }\left(1+\frac{x}{\varepsilon }+\frac{1}{\pi }\sin\left(\frac{\pi x}{\varepsilon }\right)\right), & \left|x\right|\leq \varepsilon \\ 1, & x>\varepsilon \\ 0, & x<-\varepsilon \end{array}\right. \end{array}$$

where $\delta_{\varepsilon }$ is the derivative of $H_{\varepsilon }$, i.e., $H_{\varepsilon }^{\prime }=\delta_{\varepsilon }$.

The energy ${ℜ}_{p}\left(\phi \right)$ is the distance regularization term, which is used to avoid the re-initialization and its induced numerical errors. With the Dirac delta function δ, the energy $L_{g}\left(\phi \right)$ computes the line integral of the function *g* along the zero level contour of ϕ. The energy functional $A_{g}\left(\phi \left(x,y\right)\right)$ computes a weighted area of the region $\Omega_{\phi }^{-}≜\left\{x:\phi \left(x\right)<0\right\}$. For the special case g=1, this energy is exactly the area of the region $\Omega_{\phi }^{-}$.

A preferable potential function *p* for the distance regularization term ${ℜ}_{p}$ is a double-well potential. Here, we provide a specific construction of the double-well potential $p_{2}\left(s\right)$ as

(9) $$\begin{array}{c} p_{2}\left(s\right)=\left\{\begin{array}{cc} \frac{1}{{\left(2\pi \right)}^{2}}\left(1-\cos\left(2\pi s\right)\right), & ifs\leq 1\\ \frac{1}{2}{\left(s-1\right)}^{2}, & ifs\geq 1. \end{array}\right. \end{array}$$

This potential $p_{2}\left(s\right)$ has two minimum points at s=0 and s=1.

This energy functional (2) can be minimized by solving the following gradient flow:

(10) $$\begin{array}{c} \frac{\partial \phi }{\partial t}=\mu div\left(d_{p}\left(\left|\nabla \phi \right|\right)\nabla \phi \right)+\lambda \delta_{\varepsilon }\left(\phi \right)div\left(g\frac{\nabla \phi }{\left|\nabla \phi \right|}\right)+\alpha g\delta_{\varepsilon }\left(\phi \right) \end{array}$$

where div(·) is the divergence operator and $d_{p}$ is a function defined by

(11) $$\begin{array}{c} d_{p}\left(s\right)≜\frac{p^{\prime }\left(s\right)}{s}. \end{array}$$

### 2.2. The Proposed Model

Ultrasound imaging often struggles with strong echoes and sound attenuation, leading to noisy images with blurred boundaries. This makes it challenging to segment the contours of specific tissues or organs based only on the grayscale image data. Additionally, the issue of scan conversion directly impacts the quality of ultrasound images and could exacerbate the challenges in segmenting contours based on grayscale image data [21,22]. To improve segmentation accuracy, it is crucial to integrate both imaging and prior information about the tissues and organs. Incorporating prior knowledge such as shape, appearance and location into the segmentation model can enhance its robustness and accuracy, resulting in anatomically correct segmentations. In the medical field, such prior knowledge is readily accessible due to standard patient positioning and the uniformity of human body structure.

#### 2.2.1. Shape Prior

**Figure 1:**
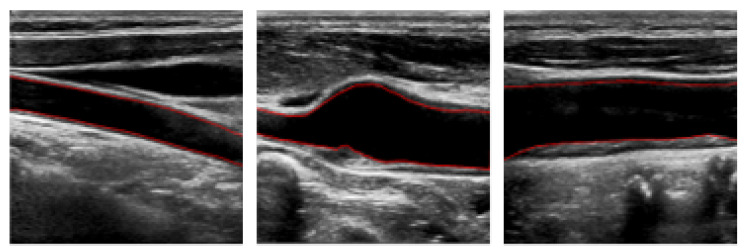
Ultrasound images of carotid artery. The red lines represent the lumen walls.

As shown in Figure 1, the longitudinal section of the carotid artery ultrasound image shows different trends in the direction of blood vessels. Based on these trends, we propose a level set segmentation algorithm based on various shape priors.

Let Ω be the image domain, and consider the image *I* as a function on Ω. Given a level set function ϕ:Ω→R, we use the signed distance function to describe the shape and integrate it into the segmentation model. When the prior shape is a known shape, the shape constraint $E_{shape}\left(\phi \right)$ can be represented as $E_{shape}\left(\phi \right)=d^{2}\left(\phi ,\phi_{0}\right)$, where ϕ and $\phi_{0}$ are the signed distance function of the target shape and the prior shape, respectively [23,24,25,26]. $E_{shape}\left(\phi \right)$ is the measure of similarity between the prior shape and the target shape, it can be written

(12) $$\begin{array}{c} E_{shape}\left(\phi \right)=d^{2}\left(\phi ,\phi_{0}\right)=\beta \int_{\Omega }{\left(H\left(\phi \right)-H\left(\phi_{0}\right)\right)}^{2}\;dx, \end{array}$$

We will use the shape prior term as the regularization term to guide the curve evolution and ensure the segmentation accuracy of the final contour.

#### 2.2.2. The Proposed Segmentation Model with Shape Constraint

Based on the general LSF Equation (2), we propose the following model

(13) $$\begin{array}{cc} E(\phi )\; & =E_{DRLSE}\left(\phi \right)+E_{shape}\left(\phi \right),\\ \; & =\mu {ℜ}_{p}\left(\phi \left(x,y\right)\right)+\lambda L_{g}\left(\phi \left(x,y\right)\right)+\alpha A_{g}\left(\phi \left(x,y\right)\right)+\beta \int_{\Omega }{\left(H\left(\phi \right)-H\left(\phi_{0}\right)\right)}^{2} \end{array}$$

where $E_{DRLSE}\left(\phi \right)$ is the energy of the DRLSE. If the initial contour is placed outside the object, then α>0, so that the zero horizontal contour will shrink during the evolution of the level set; If the initial contour is placed inside the object, then α<0 to unfold the contour. Given that, the corresponding evaluation model can be expressed as follows

(14) $$\begin{array}{c} \frac{\partial \phi }{\partial t}=\mu div\left(d_{p}\left(\left|\nabla \phi \right|\right)\nabla \phi \right)+\lambda \delta_{\varepsilon }\left(\phi \right)div\left(g\frac{\nabla \phi }{\left|\nabla \phi \right|}\right)+\alpha g\delta_{\varepsilon }\left(\phi \right)+2\beta \left(H\left(\phi \right)-H\left(\phi_{0}\right)\right) \end{array}$$

The model in (14) can be implemented with following iteration scheme

(15) $$\begin{array}{c} \frac{\phi^{t+1}-\phi^{t}}{\Delta t}=L\left(\phi^{t}\right) \end{array}$$

where $L(\phi^{t})$ is the approximation of the functional derivative $\frac{\partial E}{\partial \phi }$ at $\phi =\phi^{t}$ using finite difference scheme, Δt is the time step which limited by the Courant–Friedrichs–Lewy Condition [27] to avoid oscillations occurred.

#### 2.2.3. Shape Prior Generation

Three critical aspects must be considered when implementing shape-prior-based curve evolution models for medical imaging, particularly in vascular imaging. Firstly, this step involves accurately representing the vascular structure, which can be challenging due to the vessels’ unique and irregular nature. Anatomical knowledge is crucial here to ensure the model precisely captures the irregularities in the lumen shape. Secondly, selecting a shape prior formula that can handle the irregular and complex nature of blood vessel lumens is essential. This formula should enable the model to generalize effectively across various shapes and sizes of lumens, enhancing its applicability. Thirdly, consideration involves selecting a suitable contour method to integrate with the shape prior. Our method must effectively work with the shape priors to delineate the inner membrane of the lumen accurately.

Addressing these points involves initially obtaining a clear understanding of the vascular shape prior, which is challenging. Given the unique and irregular shapes of blood vessel lumens, utilizing irregular bands as shape priors could effectively capture the intricate details of the lumen’s inner membrane.

The shape prior in this article is generated fully automatically. This region is obtained by extending the lumen’s narrowest radius upward and downward from its approximate centerline (It will be described in detail in Section 2.2.4). Our method operates independently without manual input, automatically adjusting based on the characteristics of the lumen.

#### 2.2.4. Automatic Initialization

It is essential to select a suitable initial contour conveniently and efficiently because of its significance to segmentation accuracy. The region that extends out of the approximate centerline of the lumen serves as an initialization of the active contour, as the internal position of the centerline ensures that the contour starts from this region. The lumen’s shape allows the initial contour to be derived using simple methods. Since the position of the lumen wall varies across each two-dimensional image, it is time-consuming and tedious to adjust the initial contour of each image manually. To streamline this process, we propose a fully automated method to generate the initial contour. This approach significantly reduces labor costs, saves time and produces an initial contour that accurately matches the lumen’s shape.

**Figure 2:**
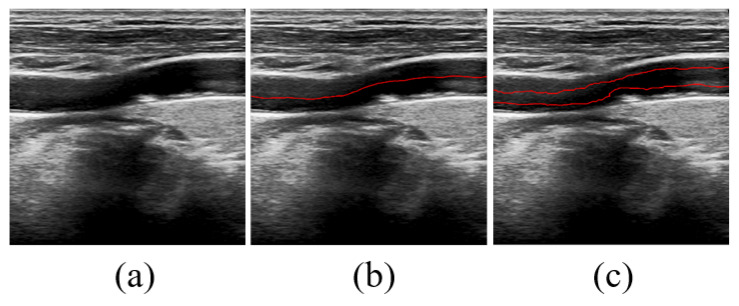
Generation process of the shape prior. (**a**) The original image. (**b**) Center line. (**c**) Shape prior.

To generate the initial contour in an image, we apply the Otsu thresholding method to perform a global threshold rough segmentation. Then, we identify the lumen region by detecting the largest connected region. Subsequently, we use morphological operators to process the boundaries extracted from the lumen region, obtaining a rough detection of the lumen wall. On the one hand, based on the rough segmentation of the lumen wall, we erode the mask of the lumen wall to 20 structural elements through morphological manipulation to obtain the mask of the initial contour, and then we obtain the initial profile. On the other hand, we determine the position of the lumen’s centerline by rough segmentation. The approximate centerline is then adjusted upwards and downwards by a certain distance, considering the narrowest distance between the near and far walls of the lumen. Finally, by combining these adjustments, we obtain the initial contour. The automatic initialization generation process is shown in Figure 2.

## 3. Experiments Results

### 3.1. Datasets

In this paper, we evaluate our algorithm using two private datasets. The detailed introduction is as follows:

(1) This dataset originates from Nanjing Brain Hospital and comprises a randomly selected collection of 149 unique longitudinal carotid artery ultrasound images. These images were collected over a period spanning from September 2019 to March 2024. To ensure the accuracy and quality of the data, all images were captured by two experienced experts in the field.
(2) This dataset, sourced from Nanjing Drum Tower Hospital, consists of 98 distinct longitudinal ultrasound images of the carotid artery, randomly selected from studies of 24 patients. These images were gathered between October 2023 and December 2023. To ensure high-quality data, all imaging was also performed by two experienced experts.

The method for manually delineating the lumen boundary involves a two-step expert evaluation. Initially, an expert with over ten years of experience interpreting ultrasound images places points along each image’s far and near walls to indicate the lumen’s boundary. Subsequently, another expert, boasting over 5 years of experience, manually connects these points with a curve to outline the lumen’s contour on the image, thereby accurately establishing the boundary of the lumen. This meticulous process ensures precise and reliable boundary delineation.

### 3.2. Evaluation Metrics

For quantitative evaluation of the segmentation performance of lumen segmentation, we applied five commonly used evaluation metrics, namely the Dice similarity coefficient (Dice), Hausdorff distance (HD), sensitivity value, specificity value and Intersection Over Union (IOU) value which can be calculated as follows.

(1) Dice coefficient

(16) $$Dice\left(X,G\right)=\frac{2|X\cap G|}{\left|X|+|G\right|}$$

(2) Hausdorff distance

(17) HD(X,G)=max(h(X,G),h(G,X))

where

(18) $$h\left(X,G\right)=\max_{x\in X}\min_{y\in G}d\left(x,y\right)$$

(3) Sensitivity value

(19) $$sen\left(X,G\right)=\frac{\left|X\cap G\right|}{\left|G\right|}$$

(4) Specificity value

(20) $$spec\left(X,G\right)=\frac{1-|X\cup G|}{1-|G|}$$

(5) IOU

(21) $$IOU\left(X,G\right)=\frac{\left|X\cap G\right|}{\left|X\cup G\right|}=\frac{TP}{TP+FP+FN}$$

where *X* and *G* denote the segmentation result and ground truth mask, respectively, TP represents the number of true positives, FP represents the number of false positives and FN represents the number of false negatives.

Thus, higher scores indicate better segmentation performance for Dice, sensitivity, specificity and IOU. For HD, lower scores indicate better segmentation performance.

### 3.3. Experimental Details

In this section, the experimental results of the proposed model and their comparison with existing models are presented in detail. All methods are processed using a laptop assembled by Lenovo in Beijing of China, with a 64-bit Windows 10 Enterprise, core i7-10710U processor and 16 GB RAM, using the Matlab2020a. According to the characteristics of the ultrasound image of the carotid artery in the experiment and our experiences, the parameters in the proposed model are fixed as follows: Δt=5, λ=6, α=−3.5, β=0.5.

### 3.4. Experimental Results

In this section, the experimental results of the proposed model are presented in detail. The proposed method has been compared with other methods regarding how precisely they segment lumens on ultrasound images of the carotid artery to evaluate the performance. An existing method named the CV model was chosen as a comparison. In addition, to highlight the validity of the shape prior, we have performed a series of ablation experiments in which we have removed the shape prior from the energy functional (13) and used distance regularization term information and data item information of images. The comparison experiments are performed on 247 annotated images, and the average Dice value, sensitivity value, Hausdorff distance, specificity value, IOU value of CV, DRLSE and proposed model are shown in Table 1.

**Figure 3:**
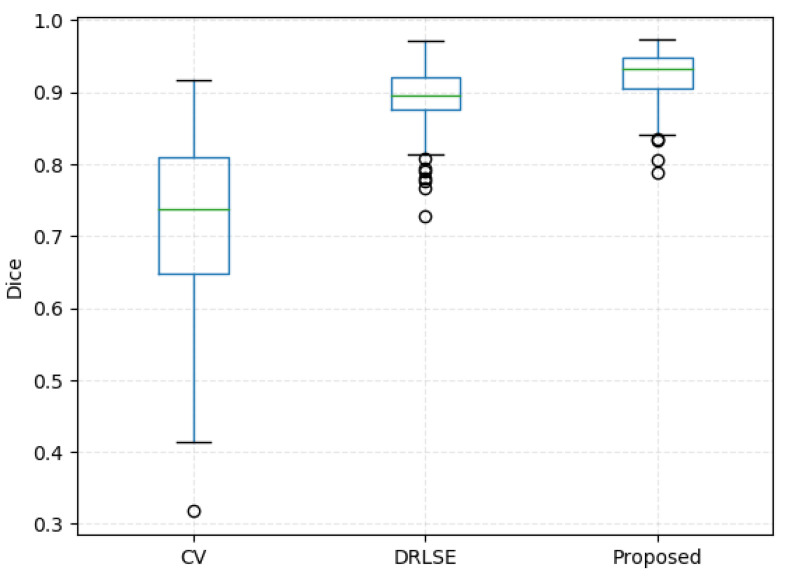
Comparison of the three methods on Dice metric.

Without incorporating shape prior information, the performance drops on 247 annotated images, leading to a decrease of 3.08% in Dice, 5.59% in sensitivity, 1.22% in specificity and 5.14% in IOU and an increase of 3.36% in HD. It can be observed that the model without the shape prior information leads to the worst performance, indicating that shape prior information is essential to obtain performance improvements. Figure 3 shows the distribution of the segmentation performance of different methods.

**Figure 4:**
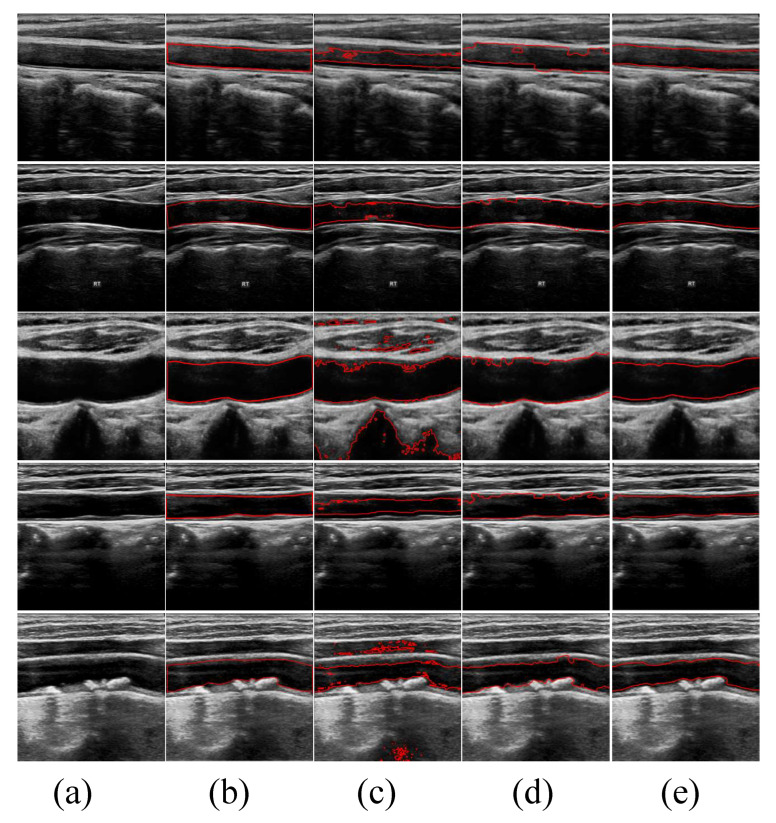
Comparison results in ultrasound images. (**a**) The original images. (**b**) Ground truth labeled by experienced physicians. (**c**–**e**) are the segmentation results of CV, DRLSE and the proposed model, respectively.

In Figure 4, we show the qualitative segmentation results of three different methods. By incorporating anatomical knowledge of the lumen, we added shape prior constraints to the traditional segmentation methods. These constraints guide and restrict the evolution of the active contours, aiming to achieve more accurate segmentation results. The first two columns are the original images and the corresponding ground truth labeled by experienced physicians. The third, fourth and fifth columns are the segmentation results of CV, DRLSE and our model, respectively. It is clearly visible that the CV model yields the poorest segmentation results, with all outcomes heavily impacted by noise. Particularly in the third image, the CV model segments almost every boundary within the image. Although the DRLSE model performs better than the CV model, its evolution curves oscillate significantly in the presence of substantial noise around the lumens, preventing accurate evolution towards the precise vessel boundaries. In contrast, our model consistently achieves better segmentation results for these types of noisy images.

### 3.5. Sensitivity Analysis

In this section, we further discuss the setting of optimal parameters of the proposed model. Four sets of parameters were randomly generated within the selected parameter neighborhood in the experiment using the pseudorandom generator. Specifically, the parameters Δt, λ, α and β were randomly sampled from [3, 7], [3, 9], [−6.5, −0.5] and [−0.5, 1.5], respectively. The experimental comparison is illustrated in Table 2. Therefore, we validated the local optimality of the parameters used in our experiments.

## 4. Discussion

### 4.1. Superiority of the Proposed Method

This paper proposes a variational model based on shape priors for segmenting the lumens in carotid artery ultrasound images. The shape priors effectively address common image issues such as speckle noise, blurred boundaries and artifacts, guiding the active contours to evolve to the actual boundaries of the vessels. The method for generating shape priors based on the centerline described in the paper is fully automatic and capable of adaptively generating priors for vessels of different shapes. Therefore, Our method represents a fully automated approach to segmenting the lumens in carotid artery ultrasound images. Although numerous methods exist for segmenting the lumens, only some can accurately segment walls in low-quality images. In our method, the designed adaptive shapes accurately represent different vessel shapes, guiding the active contours to the actual boundaries without being affected by noise artifacts, blurred boundaries or partial boundary loss. Experiments conducted using the proposed model achieved an average Dice coefficient of 92.38% for the segmentation results, demonstrating its effectiveness and precision in accurately delineating the lumens in carotid artery ultrasound images.

Compared to traditional model-based variational methods, supervised learning approaches are driven by data and can deliver precise segmentation results [28,29]. Xie et al. [28] used a U-Net convolutional neural network to segment the lumen in 2156 2D ultrasound images, achieving a 94.3% 10-fold cross-validation accuracy. Huang et al. [29] proposed a Boundary Division Network (BDNet) to address segmentation errors at discontinuous lumen boundaries and dark boundaries. They designed a feature extraction module to capture and fuse multi-scale features and features from different receptive fields to tackle the issues of inaccurate segmentation at dark and discontinuous boundaries. Additionally, they employed a new loss function to optimize the model, mitigating the impact of class imbalance on model performance and resulting in smoother, more precise boundaries. Validation on 1548 images achieved a segmentation accuracy of 65.6%, with the model showing advantages over popular deep learning methods in terms of both model efficiency and segmentation accuracy.

However, the effectiveness of the above methods is heavily reliant on the volume and quality of the data. Data-driven segmentation methods generally demand a large number of training samples, each accurately annotated in areas of interest, to ensure result accuracy. This requirement makes the process labor-intensive and cumbersome, with strict data collection and database setup demands. Furthermore, the quality of images can vary widely depending on the imaging equipment used and the experience of operators, complicating the application of data-driven methods. In contrast, model-driven approaches do not require costly annotated training sets. The accuracy of their results is mainly affected by the quality of the images, and issues such as noise artifacts and blurred boundaries can influence the segmentation outcomes. Consequently, this article utilizes a vascular adaptive shape prior model to guide the segmentation contours. This model effectively overcomes challenges like noise and blurred edges, thereby achieving precise segmentation results.

### 4.2. Limitations

**Figure 5:**
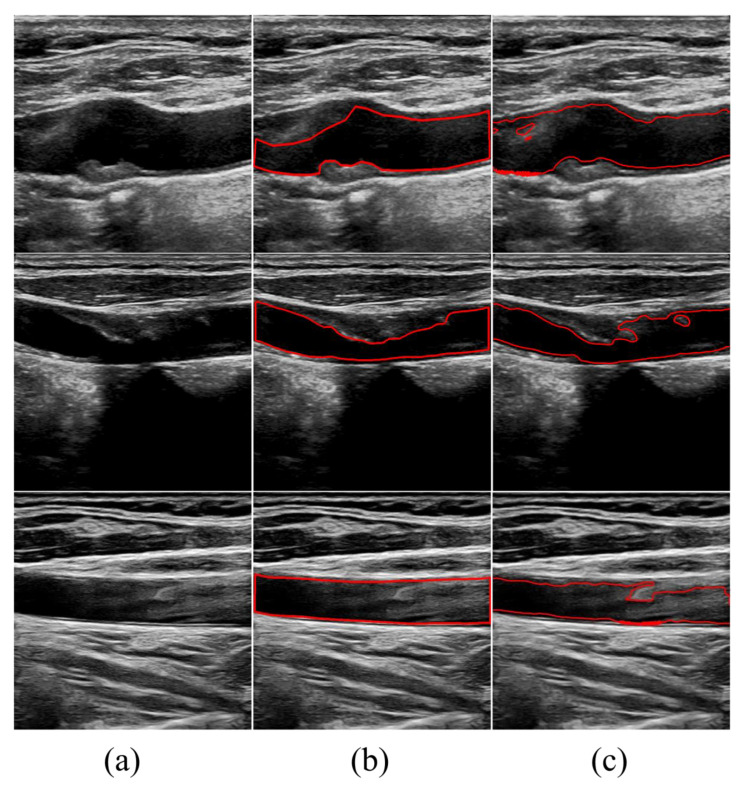
Poor segmentation results of the proposed method. (**a**) The original images. (**b**) Ground truth. (**c**) Results of the proposed method.

Although our method demonstrates the capability to segment lumens effectively, specific challenges still need to be addressed due to the imaging principles and the techniques physicians use during examinations. Some images may exhibit significant noise artifacts and severe unevenness in grayscale, which can complicate achieving better segmentation results. As illustrated in Figure 5, when there is substantial noise within the lumen, it negatively impacts the segmentation accuracy. Additionally, if the plaque within the lumen has a low grayscale value close to the noise level, it can lead to over-segmentation. Similarly, when the grayscale value of the lumen is low, the contour curve may easily cross boundaries and evolve beyond them, resulting in over-segmentation.

To address the issues of noise artifacts and uneven grayscale in carotid artery ultrasound images, improving the precision of lumen wall segmentation can be achieved through various strategies. Preprocessing techniques such as noise filtering and histogram equalization can be applied to remove noise and enhance image quality. Combining deep learning methods with traditional variational models, such as U-Net with attention mechanisms, can improve focus and segmentation accuracy in key areas. Data augmentation increases training data diversity, helping the model better cope with noise and artifacts in images. Post-processing techniques, such as morphological operations and region growing, further optimize the segmentation results.

To counter the problem of over-segmentation caused by low grayscale values of plaques and lumens in ultrasound images, several strategies can be implemented. Initially, adaptive local thresholding and local contrast enhancement techniques can be used for preprocessing to improve image quality and highlight key structures. Additionally, region-based segmentation methods and advanced deep learning technologies, such as graph convolutional networks, can be employed for more precise handling of subtle changes in the images. Morphological operations and boundary refinement techniques for post-processing help to smooth and refine the edges. Multi-scale analysis and prior knowledge of vascular structures can also be utilized to optimize the segmentation process. These comprehensive strategies help reduce over-segmentation and enhance the accuracy and reliability of segmentation.

### 4.3. Clinical Application

Our research has made significant progress in improving the segmentation accuracy of carotid artery wall in ultrasound images, and has important clinical application prospects. By integrating variational segmentation models and shape prior, we have achieved more precise segmentation even in low-quality images. This technology not only aids in early diagnosis of atherosclerosis and assessment of cardiovascular risks by analyzing wall thickness and morphology, but also plays a crucial role in stroke prevention. Accurate segmentation is vital for early diagnosis and monitoring of diseases like atherosclerosis, enabling precise evaluation of lesion size and morphology, enhancing diagnostic accuracy and facilitating personalized treatment planning, thereby providing reliable support for clinical decision-making. Our study introduces innovative methods to the field of medical image analysis, promising practical health benefits for future clinical practice.

## 5. Conclusions

In summary, this paper introduces a segmentation algorithm for the lumens in carotid artery ultrasound images based on shape priors. This algorithm adaptively generates shape prior information, which constrains and guides the contour evolution until precise segmentation of the lumens is achieved. The efficacy of the proposed model was confirmed using 247 carotid artery ultrasound images, with experimental results showing an average Dice coefficient of 92.38%, demonstrating that this algorithm can accurately segment lumens in low-quality images, which is critically important for preventing, diagnosing and interventional treating vascular diseases.

## Figures and Tables

**Table 1:** Average Dice value, sensitivity value, Hausdorff distance, specificity value, IOU value of three methods. The arrows indicate the directions towards better performance. The bold numbers denote the best performance.

Method	Dice ↑	Sensitivity ↑	Specificity ↑	IOU ↑	HD ↓
CV	72.36 ± 10.69	85.16 ± 0.00	93.94 ± 0.00	57.75 ± 1.00	60.30 ± 64.83
DRLSE	89.30 ± 3.81	91.64 ± 2.67	98.20 ± 4.12	80.88 ± 5.23	13.88 ± 7.61
Proposed	**92.38 ± 3.34**	**97.23 ± 3.34**	**99.42 ± 3.23**	**86.02 ± 1.00**	**10.52 ± 10.13**

**Table 2:** Average Dice value, sensitivity value, Hausdorff distance, specificity value, IOU value of the proposed models’ performance with different parameters. The arrows indicate the directions towards better performance. The bold numbers denote the best performance.

Parameters	Dice ↑	Sensitivity ↑	Specificity ↑	IOU ↑	HD ↓
Δt=5.00, λ=6.00, α=−3.50, β=0.50	**92.38 ± 3.34**	**97.23 ± 3.34**	99.42 ± 3.23	**86.02 ± 1.00**	10.52 ± 10.13
Δt=6.20, λ=7.75, α=−1.41, β=1.96	86.57 ± 8.21	74.03 ± 11.86	**99.70 ± 0.64**	73.03 ± 11.58	15.13 ± 14.58
Δt=5.80, λ=4.66, α=−4.21, β=1.15	90.50 ± 4.31	80.33 ± 7.77	99.57 ± 0.80	78.82 ± 7.50	11.20 ± 8.53
Δt=4.02, λ=3.98, α=−1.73, β=0.38	91.70 ± 3.71	82.28 ± 7.04	99.62 ± 0.50	80.82 ± 6.83	**10.19 ± 7.52**
Δt=4.40, λ=6.51, α=−5.38, β=0.40	91.52 ± 7.21	90.40 ± 3.99	97.49 ± 4.54	83.18 ± 9.96	21.88 ± 29.54

## Data Availability

Data are contained within the article.

## Abbreviations

The following abbreviations are used in this manuscript:

CV	Chan–Vese
DRLSE	Distance Regularized Level Set Evolution
M-S	Mumford–Shah
LSF	Level Set Function
HD	Hausdorff Distance
IOU	Intersection Over Union
TP	True Positives
FP	False Positives
FN	False Negatives
